# Luteolin Ameliorates Experimental Pulmonary Arterial Hypertension via Suppressing Hippo-YAP/PI3K/AKT Signaling Pathway

**DOI:** 10.3389/fphar.2021.663551

**Published:** 2021-04-15

**Authors:** Wanyun Zuo, Na Liu, Yunhong Zeng, Zhenghui Xiao, Keke Wu, Fan Yang, Biao Li, Qingqing Song, Yunbin Xiao, Qiming Liu

**Affiliations:** ^1^ Department of Cardiovascular Medicine, The Second Xiangya Hospital of Central South University, Hunan, China; ^2^ Department of Cardiology, Hunan Children’s Hospital, Hunan, China

**Keywords:** luteolin, Pulmonary arterial hypertension, hippo-yap, PI3K, akt

## Abstract

Luteolin is a flavonoid compound with a variety of pharmacological effects. In this study, we explored the effects of luteolin on monocrotaline (MCT) induced rat pulmonary arterial hypertension (PAH) and underlying mechanisms. A rat PAH model was generated through MCT injection. In this model, luteolin improved pulmonary vascular remodeling and right ventricular hypertrophy, meanwhile, luteolin could inhibit the proliferation and migration of pulmonary artery smooth muscle cells induced by platelet-derived growth factor-BB (PDGF-BB) in a dose-dependent manner. Moreover, our results showed that luteolin could downregulate the expression of LATS1 and YAP, decrease YAP nuclear localization, reduce the expression of PI3K, and thereby restrain the phosphorylation of AKT induced by PDGF-BB. In conclusion, luteolin ameliorated experimental PAH, which was at least partly mediated through suppressing HIPPO-YAP/PI3K/AKT signaling pathway. Therefore, luteolin might become a promising candidate for treatment of PAH.

## Introduction

Pulmonary arterial hypertension (PAH) is a chronic, progressive, extremely malignant disease with high mortality (Edda et al., 2019), which is characterized by continuously increased pulmonary vascular pressure and pulmonary vascular resistance, ultimately leading to right heart failure and sudden death (John et al., 2012). The diagnosis and treatment of PAH has made considerable progress in recent years. The 1, three and 5-years survival rates of patients with high-risk PAH are only 70, 25 and 6% (Kylhammar et al., 2018). However, it is still difficult to reverse the outcome and reduce the high mortality rate of PAH (Lau et al., 2017; Gamou et al., 2018; Kylhammar et al., 2018). The pathogenesis of PAH is related to abnormal pulmonary artery contraction, endothelial dysfunction, vascular remodeling, and *in situ* thrombosis (Thenappan et al., 2018). Among them, the remodeling of small blood vessels caused by the abnormal proliferation and migration of pulmonary arterial smooth muscle cells (PASMCs) plays a critical role (Quatredeniers et al., 2019). Therefore, investigating the underlying mechanism of development of PAH and new molecular targets that prevent pulmonary vascular remodeling is of great importance for treatment of PAH.

The Hippo/YAP signaling pathway plays a key role in regulation of cell migration and proliferation and tissue homeostasis (Johnson and Halder, 2014), and the main factors of the Hippo/YAP signaling pathway are upstream kinase large tumor suppressor 1/2 (LATS1/2) and downstream transcriptional activation cofactor Yes-associated protein (YAP) (Yu et al., 2015). When the Hippo/YAP signaling pathway is activated, LATS1/2, which is phosphorylated by upstream kinases, promotes the phosphorylation of serine 127 (Ser-127) of YAP, then phosphorylated YAP remains in the cytoplasm by binding to 14-3-3 phosphopeptide and is finally degraded by the proteasome. Conversely, inhibiting the activity of LATS1/2 can lead to dephosphorylation of YAP protein, which promotes its entry into the nucleus, binding to transcription factors and inducing the expression of target genes such as connective tissue growth factor (CTGF), MYC, Cysteine-rich angiogenic inducer 61 (CYR61), Cyclin D1, etc., finally promoting cell proliferation, migration and survival (Zhao et al., 2007; Johnson and Halder, 2014; He et al., 2018). It has been reported that LATS1 is inactivated and higher YAP has been detected in small remodeled pulmonary arteries from idiopathic PAH patients (Kudryashova et al., 2016). Moreover, YAP protein activation is involved in the migration and proliferation of PASMCs and the regulation of pulmonary vascular remodeling (Kudryashova et al., 2016), and studies have shown that YAP directly promotes the transcription of *Pik3cb,* which encodes catalytic subunit p110β of PI3K, through transcription enhancement association domain (TEAD), subsequently activating the PI3K/AKT pathway to promote cardiomyocyte proliferation and survival (Lin et al., 2015). Furthermore, PI3K/AKT pathway plays an important role in pulmonary vascular remodeling. Inhibition of AKT phosphorylation could impede the proliferation of PASMCs and attenuate pulmonary vascular remodeling (Teng et al., 2003; Gary-Bobo et al., 2010; Huang et al., 2017; Zhang et al., 2018).

Luteolin is one of natural flavonoid compounds with multiple pharmacological activities, including anti-inflammatory, anti-allergic, anti-tumor, anti-bacterial, anti-viral, etc.. Studies have shown that luteolin can arrest cell cycle, inhibit the proliferation of tumor cells, and reduce tumor cell migration (Imran et al., 2019; Kang et al., 2019; Feng et al., 2020; Iida et al., 2020). In addition, luteolin can also inhibit the proliferation and migration of vascular smooth muscle cells (Lang et al., 2012; Wu et al., 2018). Recently, it has been reported that luteolin can increase the degradation of YAP protein, thereby inhibiting epithelial mesenchymal transition and breast cancer cell migration (Cao et al., 2020), of which are implicated that luteolin may ameliorates pulmonary arterial hypertension via suppressing Hippo-YAP/PI3K/AKT signaling pathway. This experiment reported the protective ability of luteolin in experimental PAH, and explored its effect on the proliferation of rat primary PASMCs induced by platelet-derived growth factor-BB (PDGF-BB) via inhibiting HIPPO-YAP/PI3K/AKT signaling pathway.

## Materials and Methods

### Animals

This study was performed in strict accordance with the recommendations in Guide for the Care and Use of Laboratory Animals of the National Institutes of Health. The protocol was approved by the Animal Experiment Ethics Committee of the Second Xiangya Hospital of Central South University. Animals Male Sprague–Dawley rats (180–220g, 6–8 weeks old, specific pathogen-free) were supplied by Hunan Slake Jingda Laboratory Animal Co., Ltd. All rats were housed under 12 h light/12 h dark cycle at 22 ± 3 °C and given free access to water and food.

### Cell Culture

Male Sprague‐Dawley rats aged 6 weeks were euthanized with pentobarbital sodium (100 mg/kg i. p.). The main pulmonary artery, the left and right pulmonary artery were quickly stripped and transferred to the ultra-clean workbench. Then carefully separate the middle layer of pulmonary artery, and the PASMCs were extracted by tissue adhesion. Primary PASMCs were cultured in DMEM/F12 supplemented by 1% streptomycin, 1% penicillin, and 15% fetal bovine serum (BOVOGEN, Cat NO. SFBS) in 5% CO_2_ incubator. Cell medium was refreshed every 2–3 days and subcultured when they reached 70–80% confluence. Morphologic appearance by phase‐contrast microscopy and immunofluorescence with an anti‐α‐smooth muscle cell (α-SMA) antibody was used to identify PASMCs (Supplementary Figure S1). PASMCs between Passages three and five from different isolations were used to perform independent replicate experiments and were synchronized by serum starvation before intervention.

### Reagents

Luteolin with purity of greater than 98% revealed by HPLC analysis was purchased from Aladdin (Shanghai, China, Cat NO. L107329) for animals. Luteolin was provided from MedChemExpress (Monmouth Junction, NJ, United States, Cat NO. 491-70-3) for cell treatment. Carboxymethyl cellulose sodium was purchased from Aladdin (Shanghai, China, Cat NO. 9004-32-4). Monocrotaline (MCT) was provided by Sigma (United States, Cat NO. C2401). The Cell counting kit 8 (CCK-8) and cell cycle detection kit was purchased from DOJINDO (Shanghai, China, Cat NO. CK04 and C543). The EdU Cell Proliferation Kit with Alexa Fluor 555 was provided by Beyotime Biotechnology (Shanghai, China, Cat NO. C0075S). Nuclear and Cytoplasmic Protein Extraction Kit was provided by Beyotime Biotechnology (Shanghai, China, Cat NO. P0027). Bicin-choninic Acid (BCA) Protein Assay Kit was purchased from Cwbio Technology (Beijing, China, CW0014). *α*-SMA antibody was purchased from Servicebio (Wuhan, China, Cat NO. GB11044). *β*-Actin (4D3) monoclonal antibody was provided by Bioworld Technology (United States, Cat NO. BS6007M). PCNA and phospho-YAP (Ser127), total AKT and Phospho-Akt (Ser473) antibodies were provided by Cell Signaling Technology (United States, Cat NO. 14074, 13008, 4691 and 4060). LATS1 and YAP1 antibody was purchased from Proteintech (Wuhan, China, Cat NO. 17049-1-AP and 66900-1-Ig). Phospho-LATS1 (Thr1079) and PIK3CB antibodies were purchased from Affinity Biosciences (Cincinnati, OH, United States, Cat NO. AF7169 and DF6164). HRP-conjugated Affinipure Goat Anti-Mouse IgG (H + L) and HRP-conjugated Affinipure Goat Anti-Rabbit IgG (H + L) was purchased from Proteintech (Wuhan, China, Cat NO. SA00001-1 and SA00001-2). Cy3 conjugated Goat Anti-mouse IgG (H + L) and FITC conjugated Goat Anti-rabbit IgG (H + L) were provided by Servicebio (Wuhan, China, Cat NO. GB21301 and GB22303).

### Experimental Design

**FIGURE 1:**
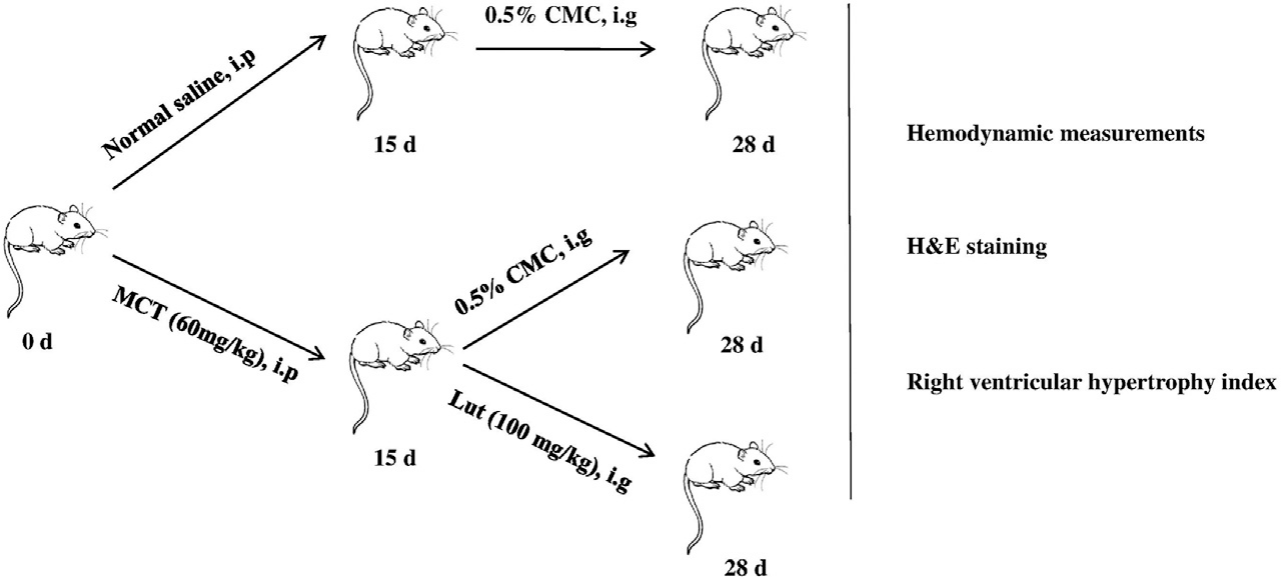
Experimental design. PAH rat models are established by single abdominal subcutaneous injection of MCT (60 mg/kg) on day 0. Subsequently, these rats of luteolin group were also was intragastrically given luteolin at 100 mg/kg/day, suspended in 0.5% carboxymethyl cellulose sodium from days 15–28. The same volume of 0.5% carboxymethyl cellulose sodium was given to the control and MCT-exposed groups from days 15–28. At the end, various experimental methods were used to evaluated the effects of luteolin. MCT, monocrotaline.

24 male rats were randomly assigned to three groups: control group (normal control, *n* = 8), model group (MCT exposure, *n* = 8), and luteolin group (MCT + luteolin, 100 mg/kg/day, *n* = 8). MCT (60 mg/kg) was administered to induce PAH by single abdominal subcutaneous injection on day 0. The control group simultaneously received normal saline on day 0. Subsequently, these rats of luteolin group were also was intragastrically given luteolin at 100 mg/kg/day, suspended in 0.5% carboxymethyl cellulose sodium from days 15–28. The same volume of 0.5% carboxymethyl cellulose sodium was given to the control and MCT-exposed groups from days 15–28. Figure 1 illustrates the animal experiment design and subsequent analysis. PASMCs were divided into four group: control group, DMSO group (as luteolin was dissolved in DMSO), model group (20 ng/ml PDGF-BB with DMSO) and luteolin group (different concentrations of luteolin).

### Hemodynamic Measurements

For right ventricular systolic pressure (RVSP) measurement, the experimental rats were anesthetized with 1% sodium pentobarbital (130 mg/kg i. p.), RVSP was measured via putting the Millar^®^ catheter (AD Instruments Inc. Colorado Springs, CO) into the right ventricle along the right internal jugular vein.

### Evaluation of Right Ventricular Hypertrophy

Following the pressure measurements, the experimental rats were euthanized, and the lungs and hearts were harvested. The hearts were divided into the right ventricle (RV) and left ventricle (LV) plus the inter-ventricular septum (S). And the right ventricular hypertrophy index (RVHI) (RVHI = weight _RV_/weight _LV+S_) were calculated.

### Histomorphometric Analysis

The rat lung tissues were fixed with 4% paraformaldehyde for 24 h for histomorphometric analysis, then embedded into paraffin, sliced into 5-μm-thick sections and subjected to hematoxylin and eosin (H.E.) staining. Randomly select 10–20 small pulmonary blood vessels with a diameter of 50–150 µm from each slice and analyze them at a magnification of 4. The two indicators that reflect pulmonary artery remodeling are calculated as follows: 1) pulmonary artery wall thickness ratio (wt%) = (outer diameter − inner diameter)/outer diameter and 2) pulmonary artery wall area ratio (WA%) = (transection area of the walls − lumen area)/transection area of the walls.

### Measurement of Cell Proliferation

CCK-8 was used to determine the effect of different luteolin doses (5–80 μM) in PASMCs proliferation. When PASMCs grown to 70–80% confluence, cells were starved for 24 h with serum free DMEM/F12 medium. After starvation, cells were pretreated with luteolin for 1 h, treated with PDGF-BB (20 ng/ml) for 24 h and incubated with CCK-8 for the last 2 h. Cell proliferation was determined by measuring the absorbance at 450 nm.

### Measurement of Cell DNA Synthesis

DNA synthesis as a measurement of cellular proliferation was obtained by EdU kit. Appropriate number of PASMCs were cultured in glass bottom dishes (Thermo Fisher Scientific, Waltham, MA, United States). After 24 h of serum-deprivation, cells were treated with luteolin (40 μM) and PDGF-BB (20 ng/ml) for 24 h. EdU incorporation was carried out according to the manufacturer’s specifications and then quantification was performed by laser confocal microscopy to randomly shoot five fields (200 cells) and count the positive rate of EdU.

### Migration Assays

PASMCs were plated into the six-well plate with five horizontal mark lines at the bottom, then cells were starved for 12 h when growing to 90% confluence. Three straight scratch was made vertically in the center of the well by 1 ml pipette tip and was photographed, then the cells were incubated with the luteolin (20 μM) and PDGF-BB (20 ng/ml). After 24 h, the scratch was photographed for comparison with the previous images. The scratch area was calculated by using ImageJ software and cell migration ratio was analyzed by (scratch area 0 h − scratch area 24 h)/scratch area 0 h.

### Western Blot

RIPA lysis buffer supplemented with complete protease and phosphatase inhibitor cocktail (100:1) was used for total protein extraction. Nuclear and Cytoplasmic Protein Extraction Kit was used for extraction of nuclear protein and cytoplasmic protein. The protein content was determined by BCA assay. Each sample was subjected to SDS-PAGE (8, 10 and 12.5%) and immunoblotted with PCNA antibody (diluted 1: 1,000), LATS1 (diluted 1:2000), p-LATS1 (diluted 1:500), YAP1 (diluted 1: 2000), p-YAP (diluted 1: 1,000), PIK3CB (diluted 1:1,000), AKT (diluted 1:1,000), p-AKT (diluted 1:2000) and *β*-actin (diluted 1:5,000) overnight at 4°C. IgG-HRP secondary antibody was incubated (diluted 1: 5,000) for 1 h at room temperature. Antibody binding signal visualization used ECL chemiluminescence kit. Finally, ImageJ software was used to attribute densitometry values to quantify the results.

### RT-qPCR

To investigate the expression of target genes, total RNA was extracted and isolated from PASMCs according to the Trizol kit instructions (Invitrogen, CA, United States). Next, the isolated RNA was reverse transcribed into cDNA using a reverse transcription kit (Thermo Fisher Scientific, MA, United States). Quantitative real-time RT-PCR was performed using the Power SYBR Green PCR Master Mix (Bio-Rad, United States). Gene expression was quantified using *β*-actin as an internal control. The PCR primers as following: LATS1: forward 5′-AGC​TCA​CTC​TCT​GGT​TGG​GA−3′ and reverse 5′-CTT​GGC​TTG​AGG​TGG​GAT​GT−3′; YAP1: forward 5′-CGT​GCC​CAT​GAG​GCT​TCG​CA-3′ and reverse 5′-TCG​GTA​CTG​GCC​TGT​CGC​GA-3′; *β*-actin: forward 5′-CAC​CCG​CGA​GTA​CAA​C-CTT​C-3′ and reverse 5′-CCC​ATA​CCC​ACC​ATC​ACA​CC-3′.

### Immunofluorescence

Cells were fixed with 4% paraformaldehyde for 30 min at room temperature, permeabilized with 0.1% triton X-100 for 20 min, and blocked with 5% BSA at room temperature for 30 min. Cell immunofluorescence staining was performed by incubation with primary antibodies (*α*-SMA, diluted 1:100; YAP, diluted 1:200) overnight, and cells were subsequently washed and incubated with fluorescent secondary antibodies at room temperature for 1 h, and finally mounted by fluorescent mounting medium with DAPI. Take pictures with laser confocal microscope.

### Statistical Analysis

All data were presented as mean ± standard deviation (SD) and performed using SPSS 24.0 Statistical Software. All *in vitro* experiments were repeated at least three times. Data were examined through one way ANOVA to determine differences between control and experimental groups. For all the statistical tests, *p* < 0.05 was considered as statistically significant.

## Results

### Luteolin Effectively Improves Monocrotaline-Induced Pulmonary Arterial Hypertension and Pulmonary Vascular Remodeling

**FIGURE 2:**
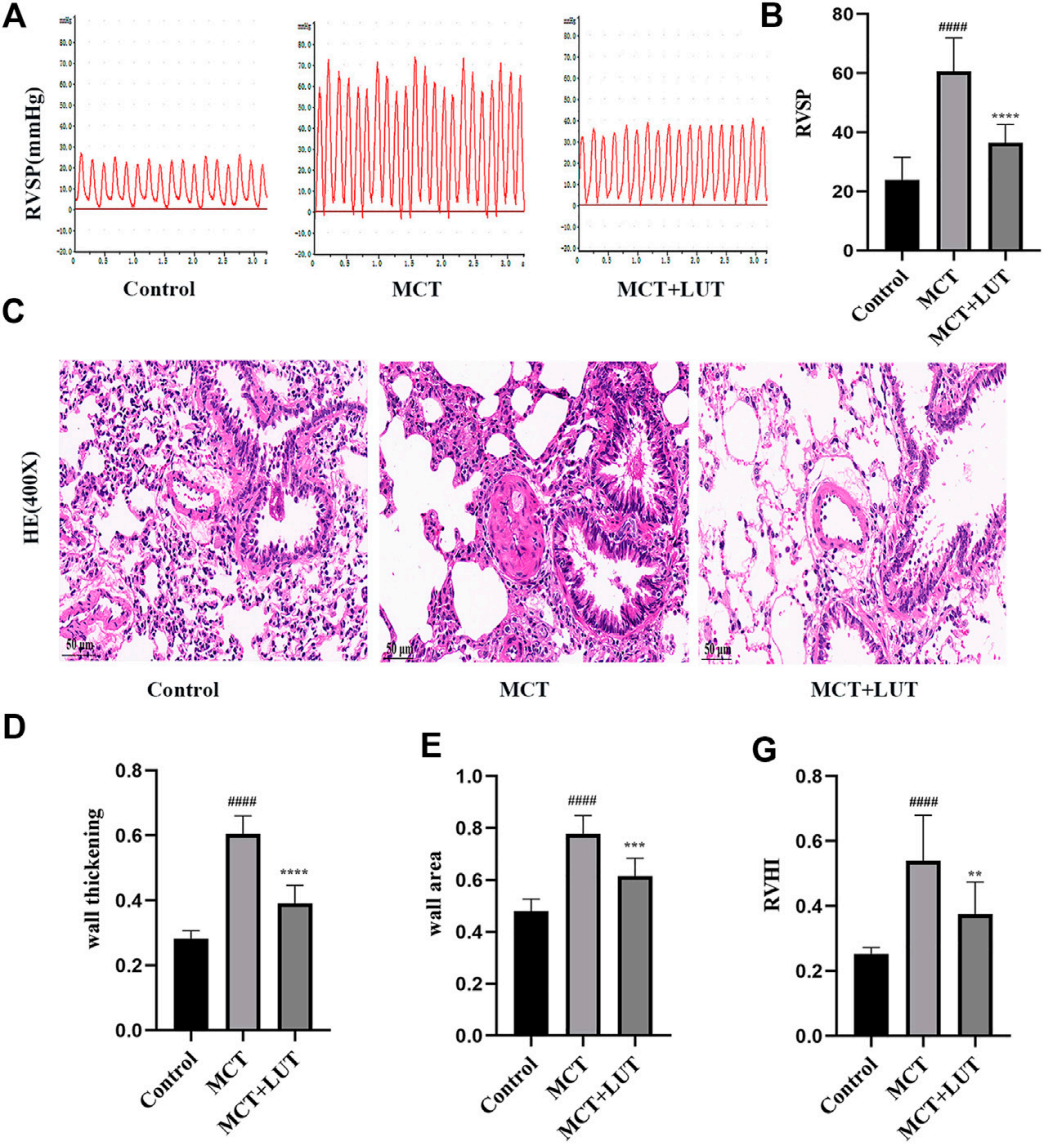
Luteolin effectively relieves MCT-induced PAH and pulmonary vascular remodeling. **(A)** Representative images of RVSP waveform (*n* = 6). **(B)** Statistical graph of RVSP (*n* = 6). **(C)** H. E. staining of pulmonary arterioles from control rats, MCT rats and MCT + LUT rats (400X) (*n* = 8) **(D)** Statistical graph of pulmonary vessel wall thickening (*n* = 8). **(E)** Statistical graph of pulmonary vessel wall area (*n* = 8). **(F)** Statistical graph of RVHI (*n* = 8). Scale: 50 μm, bars represent means ± SD, #control group *vs* MCT group, #*p* < 0.05, ##*p* < 0.01, *MCT group *vs* LUT group, *p < 0.05, **p < 0.01, ***p < 0.001, ****p < 0.0001. LUT, luteolin; MCT, monocrotaline; PAH, RVHI, right ventricular hypertrophy index; RVSP, right ventricular systolic pressure.

Compared to control group, 4 weeks after MCT injection, the rats showed a significant increase in RVSP and luteolin could effectively reverse the increase in RVSP induced by MCT (Figures 2A,B)**.** HE staining was consistent with hemodynamic results. MCT caused manifest vascular remodeling, and luteolin significantly alleviated the thickening of the pulmonary artery wall induced by MCT (Figures 2C–E). Consistent results were observed for right ventricular hypertrophy index (RV/LV + S). As shown in Figure 2F, RV/LV ± S in the MCT group was much higher than that in the control group and luteolin could reduce the increase of RV/LV ± S caused by MCT. The above results indicated that luteolin effectively relieved MCT-induced PAH and pulmonary vascular remodeling.

### Luteolin Inhibits the Proliferation of Platelet-Derived Growth Factor-BB Induced Rat Pulmonary Arterial Smooth Muscle Cells

**FIGURE 3:**
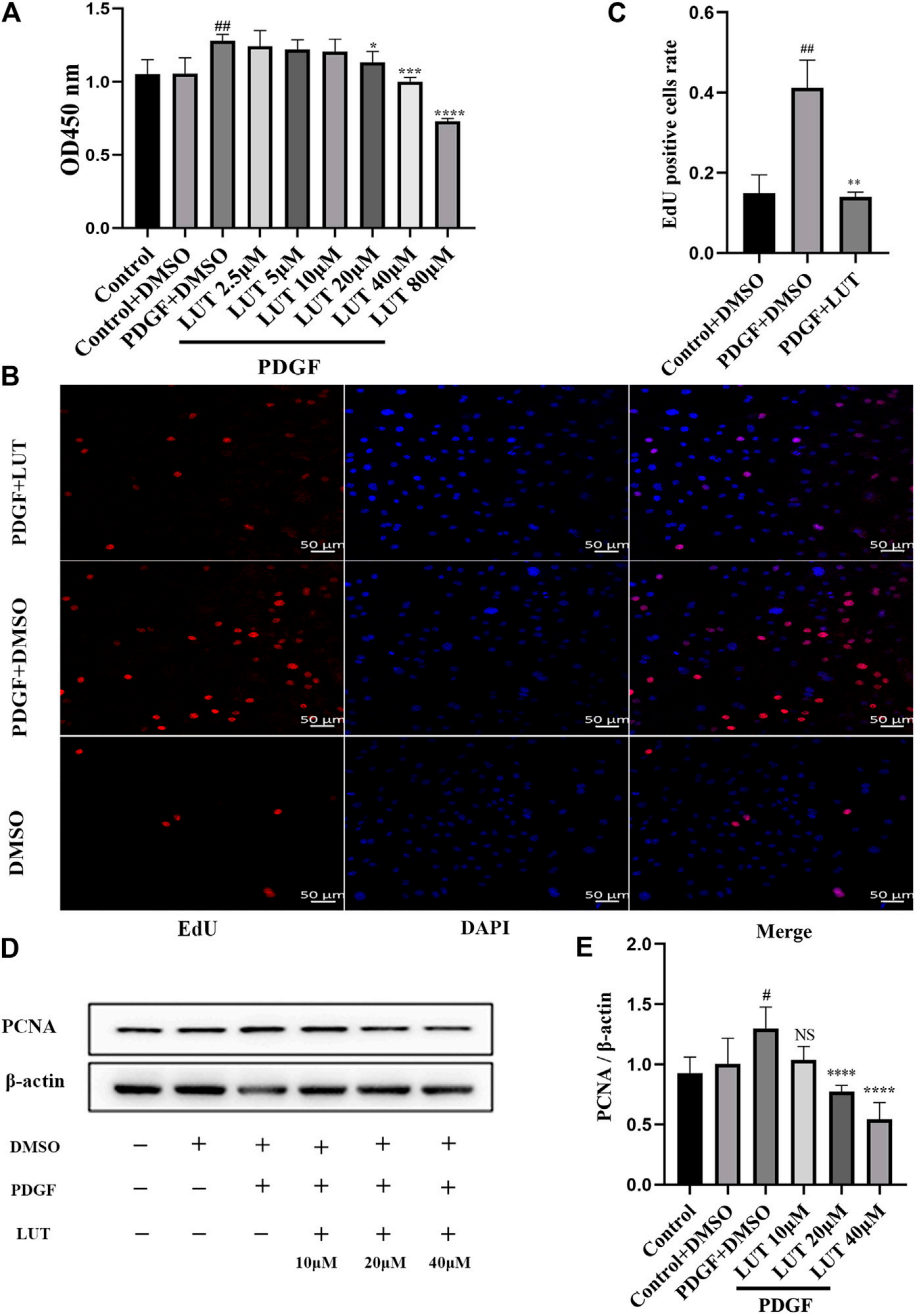
Luteolin inhibits the proliferation of rat PASMCs induced by PDGF-BB. PASMCs were cultured in 1% serum medium for 24 h, then treated with different concentrations of luteolin for 1 h before the stimulation with PDGF-BB (20 ng/ml). **(A)** Cell proliferation was examined using the Cell Counting kit eight test (*n* = 4); **(B)** and **(C)** DNA synthesis was examined using EdU, luteolin significantly inhibited the PDGF-BB induced DNA synthesis of PASMCs (*n* = 3). **(D)** and **(E)** the expression of protein level of PCNA was determined by western blot (*n* = 5). Scale: 50 μm, Bars represent means ± SD, #DMSO group *vs* PDGF + DMSO group, #*p* < 0.05, ##*p* < 0.01, *PDGF + DMSO group *vs* PDGF + LUT group, *p < 0.05, **p < 0.01, ***p < 0.001, ****p < 0.0001. DAPI, 4,6-diamino-2-phenylindole; EdU, 5-ethynyl-2′-deoxyuridine; LUT, luteolin; PASMCs, pulmonary arterial smooth muscle cells; PCNA, proliferating cell nuclear antigen; PDGF-BB, platelet-derived growth factor-BB.

Firstly, we used CCK-8 to detect the effect of luteolin on PDGF-BB induced PASMCs proliferation. Experimental results showed that PDGF-BB could effectively promote the proliferation of PASMCs, while luteolin inhibited PDGF-BB induced proliferation in a dose-dependent manner (Figure 3A). Then we used EdU incorporation experiments to evaluate the effect of luteolin on the DNA synthesis of PASMCs. The results showed that luteolin could significantly inhibit the PDGF-BB induced DNA synthesis of PASMCs (Figures 3B,C). Moreover, Western blot results showed that PDGF-BB exposure resulted in increased expression of proliferation protein PCNA, while luteolin inhibits the expression of PCNA induced by PDGF-BB in a concentration-dependent manner (Figures 3D,E). The above results indicated that luteolin inhibited PDGF-BB induced PASMCs proliferation.

### Luteolin Inhibits the Migration of Platelet-Derived Growth Factor-BB Induced Rat Pulmonary Arterial Smooth Muscle Cells

**FIGURE 4:**
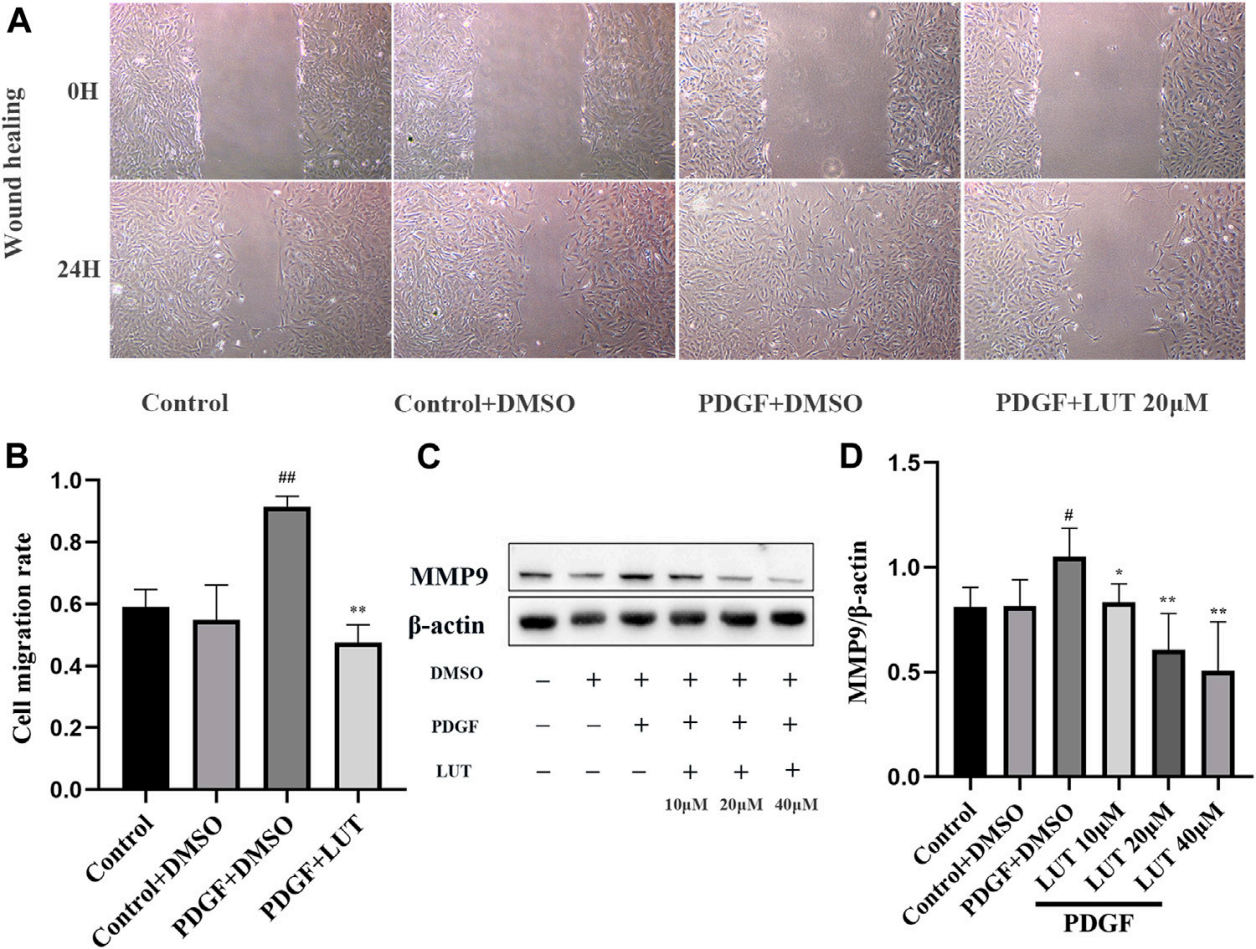
Luteolin inhibits the migration of rat PASMCs induced by PDGF-BB. PASMCs were starved for 24 h in 1% serum medium, then treated with different concentrations of luteolin for 1 h before the stimulation with PDGF-BB (20 ng/ml). **(A)** Representative images from the wound healing assay of PASMCs (40X) (*n* = 3). **(B)** Statistical graph of wound healing experiment (*n* = 3). **(C)** and **(D)** the expression of protein level of MMP9 (*n* = 3). Bars represent means ± SD, #DMSO group *vs* PDGF + DMSO group, #*p* < 0.05, ##*p* < 0.01, *PDGF + DMSO group *vs* PDGF + LUT group, *p < 0.05, **p < 0.01, ***p < 0.001. MMP9, matrix metallopeptidase 9; LUT, luteolin; PASMCs, pulmonary arterial smooth muscle cells; PDGF-BB, platelet-derived growth factor-BB.

The abnormal migration of PASMCs also plays a key role in pulmonary vascular remodeling. *In vitro*, scratch healing experiment was used to evaluate the effect of luteolin on PDGF-BB induced migration of PASMCs. PDGF-BB exposure resulted in an increase in PASMCs migration, which was restrained by Luteolin (Figures 4A,B). Notably, luteolin significantly reversed the increase in MMP9 expression induced by PDGF-BB with a dose-dependent manner (Figures 4C,D).

### Luteolin Inhibits the Increase of Transcription and Protein Expression of Key Molecules in Hippo/Yes-Associated Protein Signaling Pathway Induced by Platelet-Derived Growth Factor-BB

**FIGURE 5:**
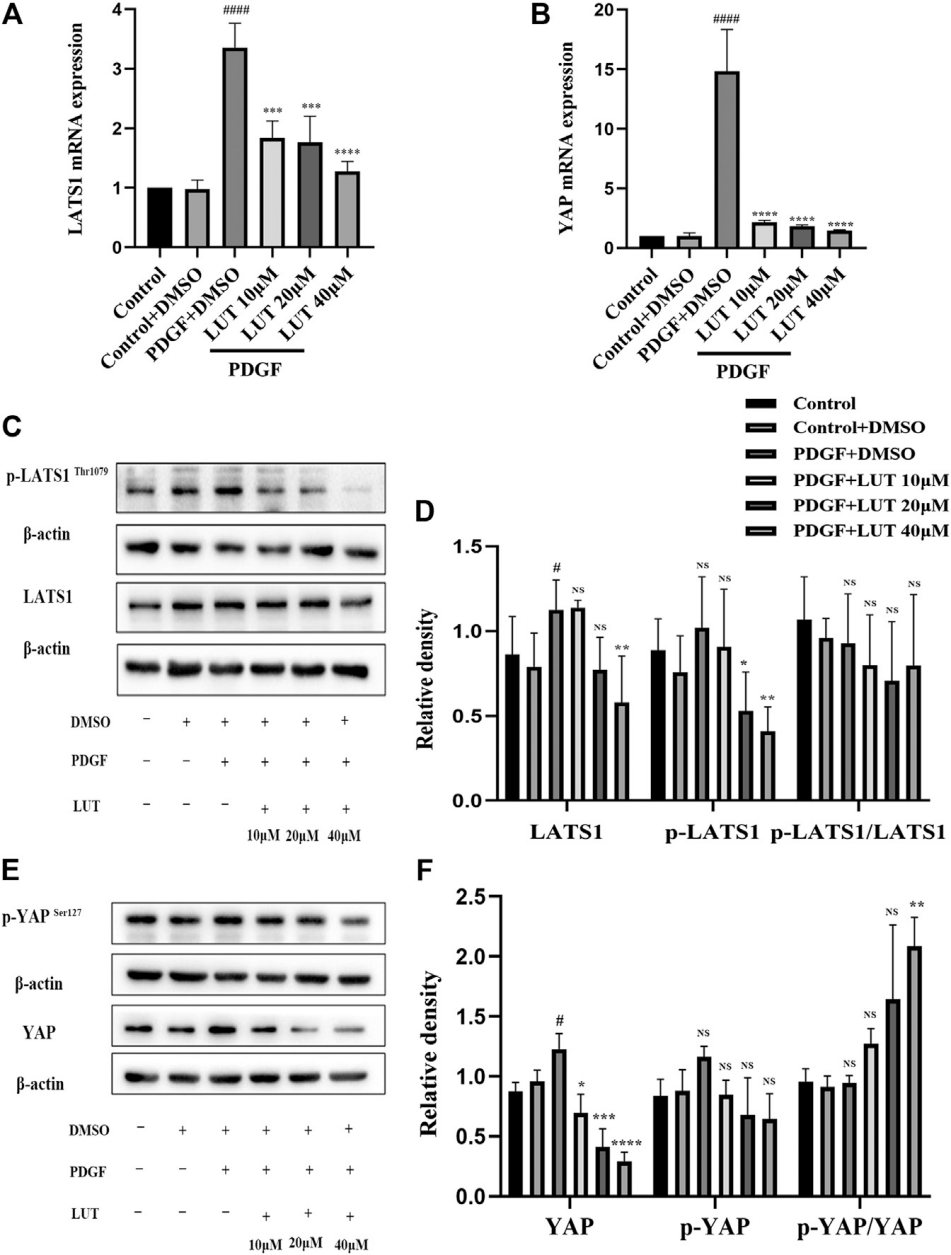
Luteolin inhibits the increase of mRNA and protein expression of key molecules in Hippo/YAP signaling pathway induced by PDGF-BB. PASMCs were cultured in 1% serum medium for 24 h, then treated with different concentrations of luteolin for 1 h before the stimulation with PDGF-BB (20 ng/ml). **(A)** mRNA level of LATS1 (*n* = 3). **(B)** mRNA level of YAP (*n* = 3). **(C)** and **(D)** the expression of protein level of LATS1 and phosphorylated LATS1 (*n* = 5). **(E)** and **(F)** the expression of protein level of YAP and phosphorylated YAP (*n* = 3). Bars represent means ± SD of *n* = 3–6, #DMSO group *vs* PDGF + DMSO group, #*p* < 0.05, ##*p* < 0.01, ###*p* < 0.0001, ###*p* < 0.0001, ####*p* < 0.00001, *PDGF + DMSO group *vs* PDGF + LUT group, *p < 0.05, **p < 0.01, ***p < 0.001, ****p < 0.0001, NS indicates no significance. LATS1, large tumor suppressor one; LUT, luteolin; PASMCs, pulmonary arterial smooth muscle cells; PDGF-BB, platelet-derived growth factor-BB; YAP, yes-association protein.

After PDGF-BB stimulation, the mRNA and protein level of LATS1 and YAP were significantly increased, which could be inhibited by luteolin (Figures 5A–F). Furthermore, we explored the expression of phosphorylated LATS1 and YAP under PDGF-BB stimulation and luteolin treatment. The experimental results showed that phosphorylated LATS1 and YAP and total LATS1 and YAP had a similar trend, but the degree of change was not as obvious as that of total LATS1 and YAP and luteolin increased the ratio of p-YAP/YAP but not p-LATS1/LATS1 (Figures 5C–F).

### Luteolin Decreases Yes-Associated Protein Nuclear Localization Induced by Platelet-Derived Growth Factor-BB

**FIGURE 6:**
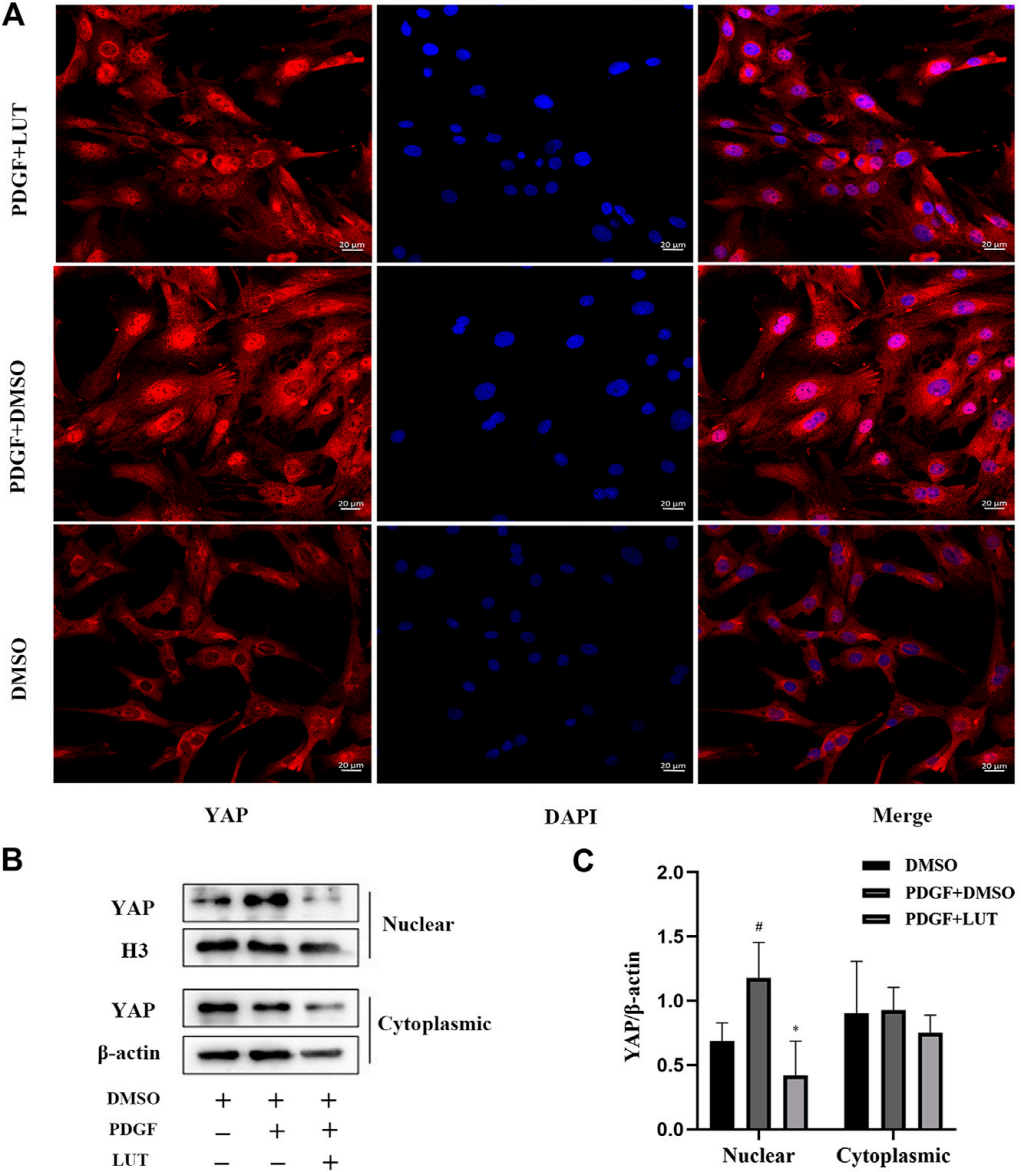
Luteolin decreases YAP nuclear localization induced by PDGF-BB. PASMCs were cultured in 1% serum medium for 24 h, then treated with luteolin (40 μM)for 1 h before the stimulation with PDGF-BB (20 ng/ml). The nuclear and cytoplasmic proteins are extracted separately and were measured by western blot analysis (A) Representative confocal microscopy images of immunofluorescence staining of YAP (red), luteolin decreased YAP nuclear localization induced by PDGF-BB (B) and (C) YAP levels in nuclear and cytoplasmic (*n* = 5). Scale: 20 μm, bars represent means ± SD, #DMSO group *vs* PDGF + DMSO group, #*p* < 0.05, *PDGF + DMSO group *vs* PDGF + LUT group, *p < 0.05. DAPI, 4,6-diamino-2-phenylindole; LUT, luteolin; PASMCs, pulmonary arterial smooth muscle cells; PDGF-BB, platelet-derived growth factor-BB; YAP, yes-association protein.

As a transcriptional co-activator, YAP regulates cell proliferation and migration mainly through its localization in the nucleus. Therefore, immunofluorescence and Western blot were used to explored the effect of luteolin on YAP nuclear localization. In the present study, PDGF-BB caused an increase in YAP nuclear localization, which were inhibited by luteolin (Figures 6A–C).

### Luteolin Inhibits PI3K/AKT Signal Pathway

**FIGURE 7:**
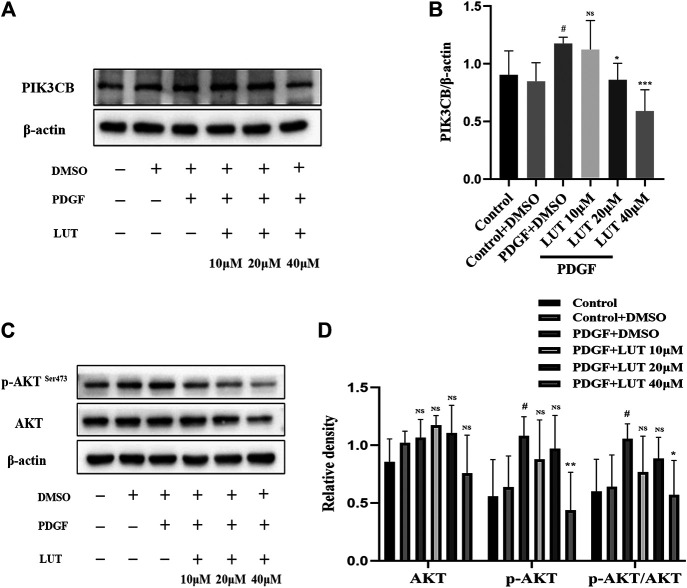
Luteolin inhibits PI3K/AKT signal pathway. PASMCs were cultured in 1% serum medium for 24 h, then treated with different concentrations of luteolin for 1 h before the stimulation with PDGF-BB (20 ng/ml). **(A)** and **(B)** The expression of protein level of PIK3CB was determined by western blot, luteolin reduced the expression of PIK3CB induced by PDGF-BB (*n* = 4). **(C)** and **(D)** The expression of protein level of total AKT and phosphorylated AKT and the ratio of p-AKT/AKT, luteolin significantly reduced the expression of phosphorylated AKT and the ratio of p-AKT/AKT induced by PDGF-BB (*n* = 5). Bars represent means ± SD of, #DMSO group *vs* PDGF + DMSO group, #*p* < 0.05, *PDGF + DMSO group *vs* PDGF + LUT group, *p < 0.05, NS indicates no significance. AKT, protein kinase B; LUT, luteolin; PASMCs, pulmonary arterial smooth muscle cells; PDGF-BB, platelet-derived growth factor-BB; PIK3CB, phosphatidylinositol 3-kinase catalytic beta polypeptide.

Hippo/YAP signaling pathway had extensive interaction with other proliferation-related signaling pathways, among which AKT is the most important candidate pathway (Kudryashova et al., 2016). Thus, we determined whether PI3K/AKT signaling pathway was also inhibited by luteolin. As anticipated, the change of PIK3CB induced by PDGF-BB was weakened by luteolin (Figures 7A,B). Luteolin significantly reduced the expression of phosphorylated AKT and the ratio of p-AKT/AKT induced by PDGF-BB (Figures 7C,D).

## Discussion

This study demonstrated that luteolin could significantly attenuate pulmonary vascular remodeling induced by MCT in rats. Simultaneously, luteolin could inhibit PDGF-BB-induced PASMCs proliferation and migration. Furthermore, this study demonstrated the protective effect of luteolin was to inhibit the expression and nuclear localization of YAP and downstream PI3K/AKT signaling pathway. This study explained the mechanism of luteolin in relieving PAH, and scientifically revealed that it may become a potential drug for the treatment of PAH.

MCT is an alkaloid from the seed crotalaria spectabilis plant, which is transformed into toxicity metabolite dehydromonocrotaline (MCTP) (Wilson et al., 1992). MCTP deposits on pulmonary capillaries and pulmonary arterioles, leading to vascular endothelial cell apoptosis, PASMCs proliferation and inhibition of PASMCs apoptosis (Nogueira-Ferreira et al., 2015). PDGF-BB is the most effective mitogenic factor in vascular smooth muscle cells, which can be excessively released by stimuli such as hypoxia, inflammation and endothelial damage. Studies have shown that PDGF-BB can stimulate the activation of downstream signal molecules by binding to its corresponding receptors PDGFR *α* and *β*, thereby triggering the proliferation and migration of vascular smooth muscle (Garat et al., 2010; Perez et al., 2010; Antoniu, 2012; Xiao et al., 2017). The expressions of PDGF and PDGFR in the lung tissues of PAH patients and animal models are significantly up-regulated, and the up-regulated PDGF and PDGFR are mainly located in pulmonary arteriole smooth muscle cells (Barst, 2005; Perros et al., 2008). These studies indicate that PDGF-BB play an important role in the proliferation of PASMCs. Therefore, this study intends to use PDGF-BB-induced PASMCs proliferation model to explore whether luteolin can improve PAH by regulating the abnormal proliferation of PASMCs.

Luteolin is a low-toxicity natural flavonoid compound that exists in many natural plants, which have been proved to have a protective effect on a variety of cardiovascular diseases, such as atherosclerosis (Ding et al., 2019), ischemia-reperfusion (Wei et al., 2018; Xiao et al., 2019), heart failure (Hu et al., 2017), and hypertension (Oyagbemi et al., 2018). Moreover, a number of studies have shown that luteolin can inhibit cell proliferation (Imran et al., 2019; Kang et al., 2019; Feng et al., 2020; Iida et al., 2020), which arouses our attention to the protective effect of luteolin on PAH. In this study, we established PAH models by injection MCT and PASMCs proliferation models by PDGF-BB treatment to study the mechanism of luteolin in improving PAH. Hemodynamic detection and histological analysis showed that luteolin could significantly reduce MCT-induced increasing in RVSP, improve pulmonary vascular remodeling and relieve right ventricular hypertrophy. Moreover, PDGF-BB induced proliferation of PASMCs could be inhibited by luteolin in CCK-8 and EdU experiments. Increased migration of PASMCs is another important cause of pulmonary vascular remodeling, which means that inhibiting the migration of PAMSCs is also important for reversing pulmonary vascular remodeling. In this study, wound healing experiments proved that luteolin significantly inhibited the migration of PASMCs induced by PDGF-BB, which suggested luteolin improved pulmonary vascular remodeling via inhibiting migration of PASMCs. Cell migration and invasion are inseparable from the protein degradation of extracellular matrix and vascular basement membrane. MMP is a zinc-dependent proteolytic enzyme of extracellular matrix, which plays a key role in the process of cell migration and invasion (Newby et al., 1994). In pulmonary arterial hypertension, the activity and expression of MMPs increase, and reducing the activity of MMPs can increase the apoptosis of pulmonary artery endothelial cells and smooth muscle cells, and improve pulmonary vascular remodeling (Lepetit et al., 2005; Okada et al., 2009). As an important member of MMPs, MMP-9 can degrade a variety of extracellular matrix and is involved in the initial proliferation of smooth muscle cells (Southgate et al., 1992). Our experimental results showed that luteolin reduced the expression of MMP-9 indued by PDGF-BB, which indicated luteolin inhibited migration of pulmonary vascular remodeling via reducing MMP-9 expression.

Pulmonary vessel remodeling caused by abnormal proliferation and migration of PAMSCs is an important pathological change of PAH (Thenappan et al., 2018), and the abnormal activation of YAP plays an important role in pulmonary vessel remodeling (Kudryashova et al., 2016). The core component of the Hippo/YAP signaling pathway is composed of a kinase chain, including Ste20-like protein kinases (MST)1/2, LATS1/2, and the downstream transcriptional coactivator YAP (Yu et al., 2015). After phosphorylation of LATS1 promotes the phosphorylation of YAP. The phosphorylated YAP is retained in the cytoplasm and is degraded by the proteasome. In contrast, dephosphorylated YAP is located in the nucleus and interacts with TEAD family transcription factors to promote target gene expression (Zhao et al., 2007; Johnson and Halder, 2014; He et al., 2018). Therefore, Hippo/YAP signal pathway is a potential target for PAH therapy. Although studies have found some YAP inhibitors, there are currently no YAP/TAZ inhibitors available clinically (Liu-Chittenden et al., 2012; Oku et al., 2015). Research by Cao et al. suggested that luteolin was a new type of YAP inhibitor (Cao et al., 2020). Our experiment results showed that luteolin could inhibit the increase in LATS1 and YAP transcription and protein expression caused by PDGF-BB. However, p-LATS1 and p-YAP had consistent changes with LATS1 and YAP, although the decrease of p-YAP had no statistical significance, p-YAP/YAP ratio increased after 40 μM luteolin treatment, which suggested that luteolin actually increased the phosphorylation efficiency of YAP. The function of YAP was determined by its localization in the nucleus or cytoplasm (Mesrouze et al., 2017), further immunofluorescence and western blot analysis showed luteolin could obviously reduce nuclear localization of YAP induced by PDGF-BB, therefore, all these finding indicated that luteolin could inhibit the activation of Hippo/YAP signal pathway by reducing nuclear localization of YAP.

Furthermore, as a transcriptional co-activator in the Hippo signaling pathway, YAP interacts with related molecules in many proliferation-related signal pathways such as PI3K/AKT, Wnt/*β*-catenin, NOTCH, which makes the Hippo/YAP pathway and other proliferation regulation-related signal pathways form a complex signal pathway network to regulate cell growth and proliferation. Among them, AKT is the most probable candidate (Kudryashova et al., 2016). YAP can directly activate the expression of Pik3cb through TEAD, promoting the transcription of PI3K and then activating AKT (Lin et al., 2015; Shen et al., 2020). PI3K/AKT signal plays an important role in regulation of cell migration, proliferation and survival (Yu & Cui, 2016). Consistent with these studies, we also observed the inhibitory effect of luteolin on the PI3K/AKT pathway activation induced by PDGF-BB stimulation.

A large number of literatures prove that luteolin is a classic flavonoid drug that inhibits the AKT pathway. And luteolin inhibits the proliferation and migration of a variety of tumor cells and vascular smooth muscle cells via inhibiting the AKT pathway (Lang et al., 2012; Ding et al., 2014; Lim et al., 2016; Yao et al., 2019; Wu et al., 2021). The latest research also has proved that luteolin is a new type of YAP inhibitor (Cao et al., 2020). At the same time, YAP promotes cell proliferation by activating the AKT pathway (Lin et al., 2015; Shen et al., 2020). YAP and PI3K/AKT signaling pathways play an important role in regulating the proliferation and migration of PASMCs (Teng et al., 2003; Kudryashova et al., 2016; Huang et al., 2017; Zhang et al., 2018). Therefore, this study chose the Hippo-YAP/I3K/AKT signaling pathway as the target pathway for luteolin to inhibit the proliferation of PASMCs. However, luteolin also has a variety of biological activities and can inhibit the activation of multiple proliferation-related pathways. Luteolin inhibits the mitogen-activated protein kinase (MAPK) signaling pathway of the downstream signaling pathway of epidermal growth factor, thereby inhibiting the proliferation of glioblastoma (Anson et al., 2018). Luteolin restrained the proliferation and migration of gastric cancer cells by inhibiting Notch signal transduction (Zang et al., 2017). In addition, Inflammation is also involved in the occurrence and development of PAH. Inflammatory factors such as interleukin 6, interleukin 21, interleukin 1β can also promote the proliferation and migration of PASMCs (Hashimoto-Kataoka et al., 2015; Prins et al., 2018; Zhang et al., 2020; Tang et al., 2021). luteolin exert anti-inflammatory effects though inhibiting the expression and secretion of a variety of inflammatory factors (Seelinger et al., 2008; Nabavi et al., 2015). Similarly, PDGF-BB can also promote cell proliferation and migration through a variety of other ways. The study of Xiao et al. proved that PDGF-BB enhanced the Warburg effect and promoted the proliferation of PASMCs through the PI3K/AKT/mTOR/HIF-1α pathway (Xiao et al., 2017). Zhang et al. proved that PDGF-BB activated the Ras homolog gene/a Rho-associated coiled coil-forming protein kinase (Rho/ROCK) pathway of PASMCs (Zhang et al., 2019). PDGF-BB activates MAPK pathway to induce the migration of retinal pigment epithelial cells (Chan et al., 2013) and the proliferation of PASMCs (Cui et al., 2016). Our research proved that luteolin restrained the proliferation of PASMCs and improved PAH at least partly through inhibiting Hippo-YAP/PI3K/AKT pathway, but whether luteolin inhibits the proliferation of PASMCs through signal pathways remains to be further studied.

## Conclusion

In summary, luteolin ameliorates experimental pulmonary arterial hypertension, and which exerts protective effects partly by reducing YAP nuclear localization and inhibiting the activation of the downstream PI3K/AKT pathway. Therefore, luteolin might be a promising candidate for PAH target medicine.

## Data Availability

The raw data supporting the conclusions of this article will be made available by the authors, without undue reservation.
