# Supplementary figures and images for: Luteolin Ameliorates Experimental Pulmonary Arterial Hypertension via Suppressing Hippo-YAP/PI3K/AKT Signaling Pathway
Source: Front Pharmacol. 2021 Apr 15;12:663551. doi: 10.3389/fphar.2021.663551 (PMC8082250; doi:10.3389/fphar.2021.663551)

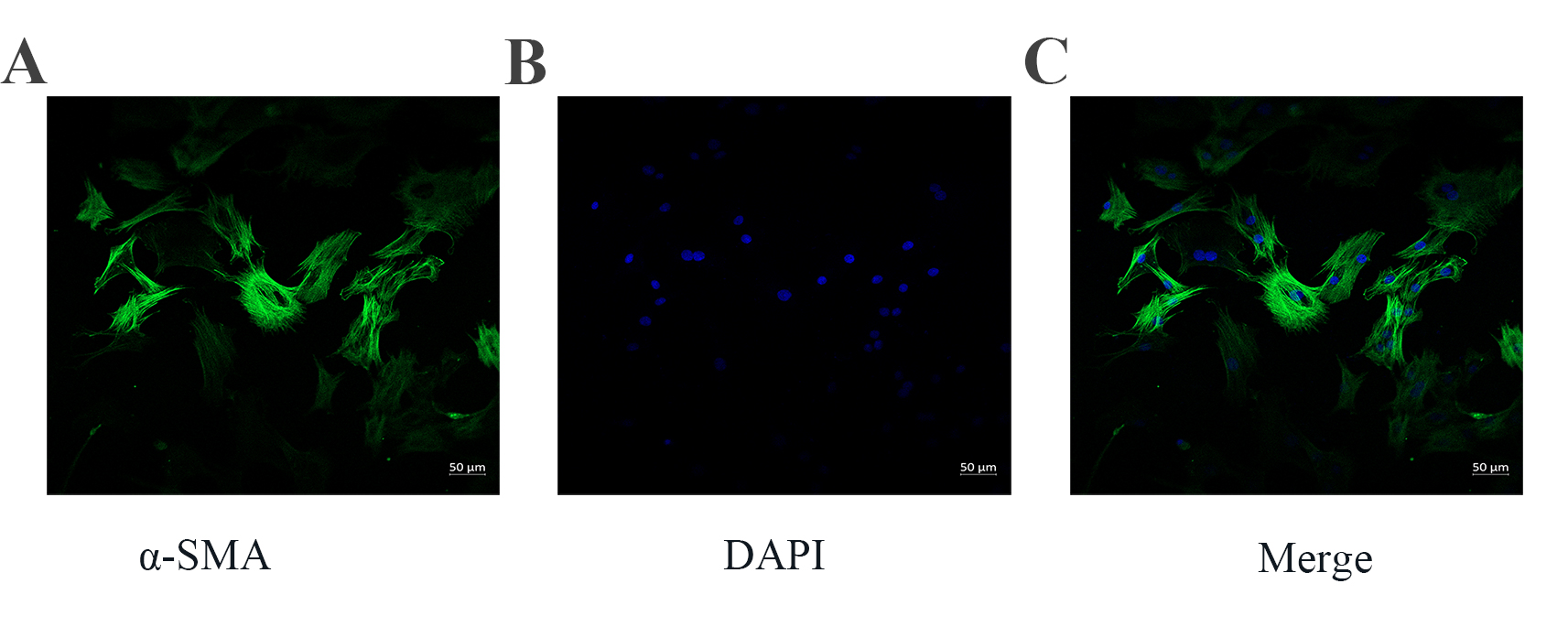

Supplement: Supplementary file 1 [file Image1.jpg]
